# A Hospital Officer's Experiences in a Nursing Home

**Published:** 1913-09-27

**Authors:** 


### HOSPITALS FROM THE PATIENTS' POINT OF VIEW.

(Contributions to this Section are Invited.)

### A Hospital Officer's Experiences in a Nursing Home.

As money was an object, I went to a nursing home in connection with one of our large hospitals, the charge for maintenance being two guineas a week. As far as I could see, the accommodation provided all that was necessary. One had not a separate room, of course, but the ward, although it contained a long double row of beds, was nevertheless very wide, too, so that one was not looking one's *vis-a-vis* out of countenance what time his screens were withdrawn. There was a good interval of space, too, on either hand. At our local infirmary they have just come in for a very handsome legacy, and are said to be employing it first of all in putting up extra beds in every ward, so as to relieve the waiting list of patients. Of course, the lesser evil is always to be chosen, and the working-classes, poor folk, have become inured to doing without much privacy. Still, a little elbow-room is a blessing.

### Fellow Patients.

As for the company I fell into, I had thought of the line—"Misery makes a man acquainted with strange bed-fellows," and so I asked the medical officer what sorts and conditions of people they commonly entertained. He replied: "We have had dukes, and we have had dustmen." Thus was Shakespeare justified. The most of the patients were, however, nearer the peerage than the scavengering department, and probably, nay necessarily, a private nursing home would contain those connected with the leisured and moneyed classes in greater proportion still; indeed, almost exclusively. Now this fact I take to be responsible for a good many of the grumbles. An habitual high standard of personal luxury is not a good preparation for the discomfort inseparable from invalidism.

There is a notion a good many people have, too, that the man who is not afraid to make a noise gets most done for him in the end. The last two visitants to nursing homes from my own neighbourhood that I have talked to were both dissatisfied. The one was a cleric. He soon left the institution and received most of his after-treatment at an hotel. But he was an exceptionally particular individual, who, if in another person's house he felt a draught, or else wanted more fresh air, would immediately go himself and close or open the window, as the case might be. He proposed, in the first instance, to stay after the operation at a brother cleric's house; but the latter, who knew his man, returned a polite "No." The other said "something was not right." He approached the senior surgeon on the subject, through a junior one, who was a friend of his. Really, a little stoicism would benefit some people. I believe, too, that doctors and nurses like uncomplaining patients, and take particular pains as regards them.

Myself, I could not take any credit in this respect, because mine was a very trifling affair, and I was up in a week. However, I was quite sick enough after the operation, and received every due attention. The up-patients had another room to go into and sit during the day. It happened to be Christmas time, too, and we had the other ward entertainments to go and look at. I was carried up some stairs and helped down again afterwards very carefully. There were some serious cases in.

I remember a Japanese, who walked to the operating theatre, firmly and bravely, with head bowed forward, probably only because he felt himself an object of general attention, to undergo, it was said, a very long and serious operation. I do not think those people in great danger complain much, any more than the ones, like myself, in no danger. It is those betwixt and between, especially if their illness takes a rather trying, chronic form, who find fault most. This is probably because they are angry, or cross; and they are cross because they are naturally apprehensive. You see the same thing in the presence of other sorts of danger. The man who feels himself out of the wood is a very amiable person; so, in another way, is the one faced by some big and unavoidable trial. But the one who is not a good companion is he who finds himself exposed to risk which is of some duration and rather more than a trifle. When certain people were tetchy they reminded me of the bad tempers shown by beginners at polo in India. To be quite plain and rather brutal, it was a case, in each instance, of anger from fear.

### Ward Noises at 5 a.m.

Perhaps if I had had an illness of medium, instead of no, severity, I might have been writing differently now; however, my sting comes at the end. What *is* the use of making poor sick people lose their beauty sleep? I know it is the same everywhere. The system of clattering about the ward at 5 a.m. probably dates from the time of the Crimean War, and arose, perhaps, as a reaction from the *régime* of Mrs. Gamp of unlamented memory. Nurses themselves have often heartily agreed with me how foolish the plan is. But I doubt whether any of them would move to change it. A little bit of appearance would have to be sacrificed, and, perhaps, the nursing staff would have to be slightly enlarged; then, too, ladies are so conservative. But I should like to see some good organiser devote his talent to this delicate question. A "he" would have less of *parti pris* in the matter than a "she." However, a Miss Buss could have solved it. May the right person, male or female, soon arise!

